# Drought Vulnerability Assessment Using Geospatial Techniques in Southern Queensland, Australia

**DOI:** 10.3390/s21206896

**Published:** 2021-10-18

**Authors:** Muhammad Hoque, Biswajeet Pradhan, Naser Ahmed, Abdullah Alamri

**Affiliations:** 1 Centre for Advanced Modelling and Geospatial Information Systems (CAMGIS), Faculty of Engineering and IT, University of Technology Sydney, Ultimo, NSW 2007, Australia; MuhammadAl-Amin.Hoque@uts.edu.au; 2 Department of Geography and Environment, Jagannath University, Dhaka 1100, Bangladesh; Naserbipu.geo2011@gmail.com; 3 Earth Observation Centre, Institute of Climate Change, Universiti Kebangsaan Malaysia, Bangi 43600, Malaysia; 4 Department of Geology and Geophysics, College of Science, King Saud University, Riyadh 11362, Saudi Arabia; amsamri@ksu.edu.sa

**Keywords:** drought, vulnerability assessment, remote sensing, GIS, spatial analysis, Australia

## Abstract

In Australia, droughts are recurring events that tremendously affect environmental, agricultural and socio-economic activities. Southern Queensland is one of the most drought-prone regions in Australia. Consequently, a comprehensive drought vulnerability mapping is essential to generate a drought vulnerability map that can help develop and implement drought mitigation strategies. The study aimed to prepare a comprehensive drought vulnerability map that combines drought categories using geospatial techniques and to assess the spatial extent of the vulnerability of droughts in southern Queensland. A total of 14 drought-influencing criteria were selected for three drought categories, specifically, meteorological, hydrological and agricultural. The specific criteria spatial layers were prepared and weighted using the fuzzy analytical hierarchy process. Individual categories of drought vulnerability maps were prepared from their specific indices. Finally, the overall drought vulnerability map was generated by combining the indices using spatial analysis. Results revealed that approximately 79.60% of the southern Queensland region is moderately to extremely vulnerable to drought. The findings of this study were validated successfully through the receiver operating characteristics curve (ROC) and the area under the curve (AUC) approach using previous historical drought records. Results can be helpful for decision makers to develop and apply proactive drought mitigation strategies.

## 1. Introduction

Droughts are climatic disasters, with varying frequencies and intensities, that extend worldwide [1,2]. The considerable effects of droughts are observed on the availability of water and on agricultural, economic, environmental and socio-economic activities [3,4,5]. Particularly, regional economic development, food and ecological security are threatened by drought [6]. On average, an estimated US $6–8 billion in economic losses are caused by drought each year worldwide, a number higher than the economic losses caused by other meteorological disasters [7]. The frequency and severity of drought have recently increased considerably in many areas of the world [8,9]. As expected, drought occurrences and the extent of these effects will be higher in the coming years due to climate change and an increase in water demand for human activities [10,11,12].

Given its geographical location and rainfall pattern, Australia is one of the driest continents [1,13,14,15]. In Australia, droughts with various intensities frequently occur, which detrimentally affect river ecosystems and agricultural activities [4,16,17]. The economy incurs enormous losses, and a humanitarian crisis is created [1,15]. Australia experienced a long and devastating drought at the beginning of the 21st century, called the millennium drought (2001–2009) [16,17,18]. According to the Bureau of Meteorology records from 1860, Australia experienced severe drought with an 18-year interval on average [18,19]. Queensland is one of the highly drought-vulnerable states in Australia [20,21,22]. Currently, the southern Queensland, particularly its southeast and southwest parts, is experiencing a severe drought that began in 2017 [2,19]. In May 2019, almost two-thirds of Queensland was declared drought vulnerable by the Queensland government. Resultantly, environmental, agricultural and socio-economic activities are affected sectors in such areas of Queensland [19,20].

Effective drought management can reduce the overall effects of droughts on people, economy and the environment [2,8,23]. The development and timely implementation of drought mitigation strategies are parts of effective drought management [9,24]. Spatial information regarding factors liable for droughts, vulnerable areas, and levels of vulnerability are essential in preparing and implementing drought strategies [25]. Drought vulnerability mapping offers a framework for identifying, processing and analysing relevant factors that trigger drought [6,26,27]. The term vulnerability explains the extent of suffering and effects on the community and environment from a particular hazard in a specific area [26,27,28]. The produced vulnerability map can also help decision makers to visualise the exact location and level of vulnerability to droughts and convey this information to respective affected sectors to reduce potential drought-related losses [29,30].

Satellite remote sensing and spatial analysis are efficient drought vulnerability mapping tools [8,31,32]. Various spatial and non-spatial data are essential for preparing the relevant spatial criteria layers that influence drought vulnerability [24,31]. Remote sensing and spatial analysis provide a suitable framework for analysing, mapping and integrating different criteria to assess drought vulnerability accurately [29,32]. Several drought vulnerability mapping approaches are available in the literature [1,8,24,31,33,34,35]. Some approaches focus on a single criterion [1,35,36], whereas others use multi-criteria [25,34,37]. Multi-criteria assessment for each category of drought can provide detailed spatial drought vulnerability information. The fuzzy analytical hierarchy process (FAHP) is one of the best techniques to assess the multi-criteria of each category of drought and integrate them for spatial decision making to assess overall drought vulnerability [31,32]. This technique reduces the imprecision and subjectivity in the pair-wise comparison decision-making process [36,37]. Instead of a crisp value, FAHP uses a range of value to incorporate the decision maker’s uncertainty [38]. FAHP has been widely applied in assessing the vulnerability of other natural hazards [39,40,41,42]. Several studies have also reported drought hazard analysis [25,33,34].

Several studies have been conducted on drought mapping, monitoring and management in Australia [2,4,17,19,20,22,43,44,45]. Most of these studies have focused either on meteorological [4,22,23,46] or agricultural drought forecasting and monitoring [2,16,21]. For example, Feng, et al. [19] used various remote sensing drought factors to manufacture agricultural drought in southeastern Australia. A meteorological- and soil-moisture-based drought index was developed by Mpelasoka, et al. [44] for present and future drought assessment in Australia. Deo, et al. [4] applied an effective drought index using meteorological data for monitoring drought events in Australia. Some studies have focused on hydrological drought forecasting and monitoring [17,45]. For example, Verdon and Franks [17] assessed long-term hydrologic drought risks in New South Wales using paleoclimatic data. Many studies have also used limited criteria in drought forecasting and monitoring [2,4,21,22]. For example, Dayal, et al. [43] used artificial neural networks (ANNs) for forecasting drought events using only historical climate data of two small regions (temperate and grassland) of southeast Queensland. Generally, droughts are broadly categorised into four classes: meteorological, agricultural, hydrological and socio-economic [46,47]. Hence, integrating as many criteria that influence these categories as possible is necessary for deriving comprehensive overall drought vulnerability information to support the effective drought management. However, studies related to drought vulnerability assessment integrating multiple categories of droughts with sufficient criteria are limited in Australia. Recently, Dayal, et al. [20] developed a drought risk map for southeast Queensland using fuzzy logic. They integrated vulnerability, hazard and exposure criteria and focused mostly on agricultural drought in their drought risk assessment. Although the southern Queensland region is prone to drought, no study has mapped a comprehensive drought vulnerability, integrating multiple drought category with adequate criteria using FAHP-based weighting scheme. 

The current research is designed to develop a comprehensive drought vulnerability map that combines meteorological, hydrological and agricultural drought vulnerability by applying geospatial techniques and to assess the spatial extent of drought vulnerability for southern Queensland. The objectives addressed in this research are as follows. First is to prepare a vulnerability map of meteorological, hydrological and agricultural drought applying a multi-criteria FAHP-based decision-making approach. Second is to develop a comprehensive drought vulnerability map that combines meteorological, hydrological and agricultural drought vulnerability maps to assess the degree of drought vulnerability. Third is to evaluate the generated drought vulnerability results.

## 2. Study Area

**Figure 1:**
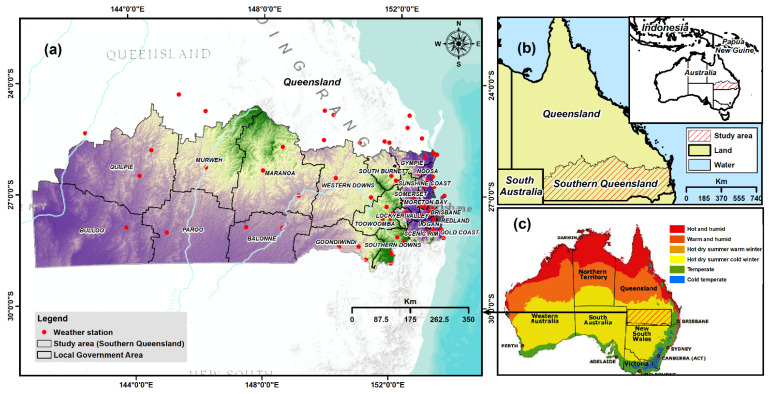
(**a**) Extent of study area outlining weather stations drawn on SRTM elevation data; (**b**) Study area in the context of entire Queensland State and Australia; and (**c**) Climatic zones of Australia.

The present study was applied in southern Queensland, Australia. The area occupies the southwest and southeast parts of Queensland (Figure 1) with a total area of 434,440 km^2^. The study region is between 24°00′–30°040′ S latitude and 142°00′–152°00′ E longitude. The total population of this region is 748,470, which is booming because of migration from overseas and other states [48]. This region is renowned for cattle grazing, horticulture, animal production and cotton farming [20]. 

Climate change, availability of water, economic burdens and population growth are fundamental challenges for sustainable agricultural activities in this region [49]. This region is also often affected by droughts that have extensive effects. Climate change and other factors are triggering these droughts directly [21]. Water supply for irrigation is disrupted frequently and continuously by droughts. A sub-tropical climate prevails in Southeast Queensland. The average temperature in the summer is 24 °C, whereas 14 °C in winter. Most rainfall occurs in the summer and autumn with 388 and 295 mm per year, respectively. Meanwhile, southwest Queensland is part of Australia’s arid inland. The average annual maximum and minimum temperatures are 28 °C and 14.5 °C, respectively. The average historical annual rainfall fluctuates between 492 and 286 mm across this region from 1879–2015.

## 3. Materials and Methods

**Figure 2:**
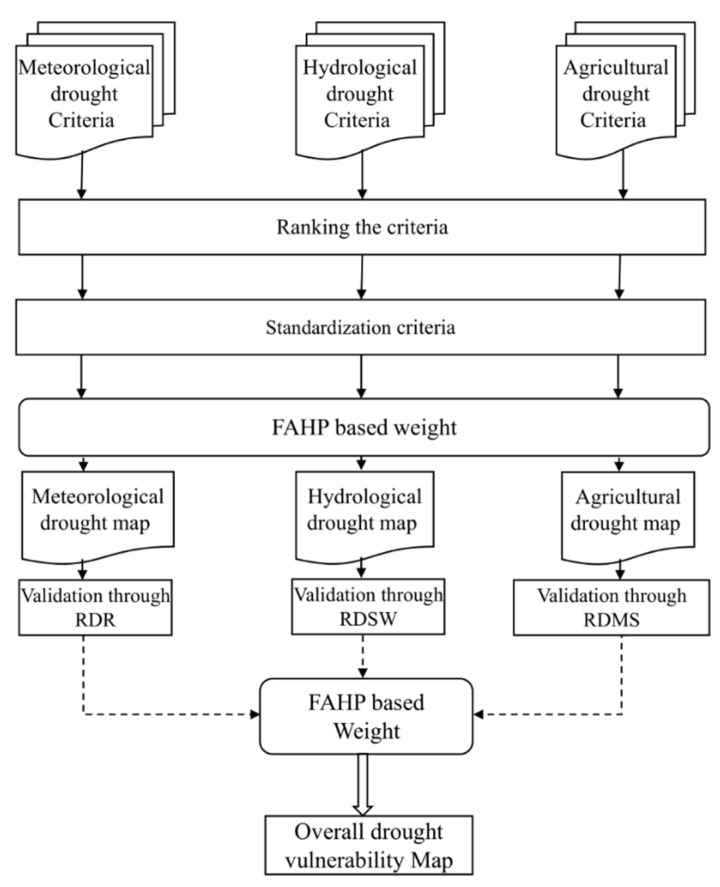
Methodological flowchart applied for assessing drought vulnerability in the present study.

A comprehensive drought vulnerability mapping approach was developed that incorporates meteorological, hydrological and agricultural drought information for southern Queensland using geospatial techniques. Thirteen criteria were selected, and their individual spatial layer was created from various data sources using geospatial techniques. FAHP was used to rank and assign the weights of the 13 criteria in producing an integrated drought vulnerability map. The methodological process is summarised in Figure 2, and their detailed descriptions are presented in the subsequent sections. 

### 3.1. Dataset and Sources

Various spatial and non-spatial data pertaining to the 13 criteria were used in this study to prepare a comprehensive drought vulnerability map. The required datasets were acquired from local, national and international organisations. The collected data were processed using remote sensing and geospatial tools to generate spatial layers for each criterion. We also used validation datasets collected from the relevant organisation and peer-reviewed published articles. These datasets, with their sources and other characteristics, are detailed in Table 1.

### 3.2. Evaluation Criteria for Vulnerability Mapping

A total of 14 criteria were chosen under three categories of drought on the basis of the literature review, data accessibility, local study context, and relevance to drought vulnerability. Following the importance of vulnerability to drought, every criterion was mapped, categorising into several alternatives to convert to spatial layers. All spatial layers were transformed into raster format with a spatial resolution of 90 m × 90 m for supporting a raster-based analysis. The natural break statistical approach was also used to classify some criteria spatial layers [30,50]. ENVI (Version 5.4) and ArcGIS (Version 10.4) geospatial software were applied to process the spatial criteria layers. The characteristics, importance, justification and mapping approaches of the influencing criteria of each category of drought are discussed in the next section.

#### 3.2.1. Meteorological Drought Vulnerability Mapping Criteria

Dry weather pattern creates a favourable condition for meteorological drought [51,52]. Several variables, such as mean annual rainfall, mean annual maximum temperature, mean annual evaporation and mean annual humidity were used for mapping meteorological drought vulnerability. The natural break statistical approach was used to classify meteorological drought criteria spatial layers [30,50].

**Figure 3:**
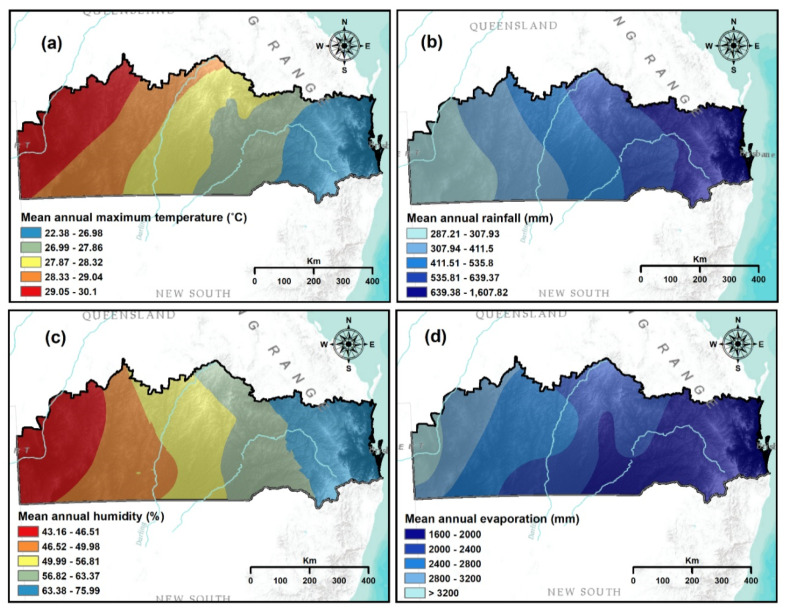
Thematic layers used for meteorological drought vulnerability: (**a**) mean annual maximum temperature; (**b**) mean annual rainfall; (**c**) mean annual humidity; and (**d**) mean annual evaporation.

Meteorological droughts are influenced by temperature, rainfall, humidity and evaporation parameters [53]. Areas prone to drought are characterised by low rainfall and humidity [30,54]. In addition, places that have a higher temperature are more exposed to drought than places characterised by low temperatures [53,55]. Moreover, evaporation is directly linked to meteorological drought. Areas with high evaporation are more vulnerable to meteorological drought because of the increased amount of water evaporation, thus making the region drier [32,53]. Meteorological drought-related criteria (precipitation, temperature and humidity) datasets were acquired from the Bureau of Meteorology of Australian government from 1970–2018 from 79 weather stations, located inside or nearby of the study area (Figure 1). The globally accepted kriging method was used [56] to interpolate the point-based datasets for producing a spatial layer of the mean annual maximum temperature, the mean annual rainfall and the mean annual humidity (Figure 3a–c respectively). The annual evaporation data were also acquired from the Bureau of Meteorology of Australian government from 1975–2005 (Figure 3d). The spatial resolution was set to 90 m for all output layers.

#### 3.2.2. Hydrological Drought Vulnerability Mapping Criteria

The scarcity of subsurface or surface water supply(i.e., in streamflows, lakes, reservoirs and groundwater) in a period is linked to hydrological drought [29,57]. Elevation, slope, surface water bodiesstream density, and surface runoff were selected as relevant criteria for mapping hydrological drought vulnerability [15].

**Figure 4:**
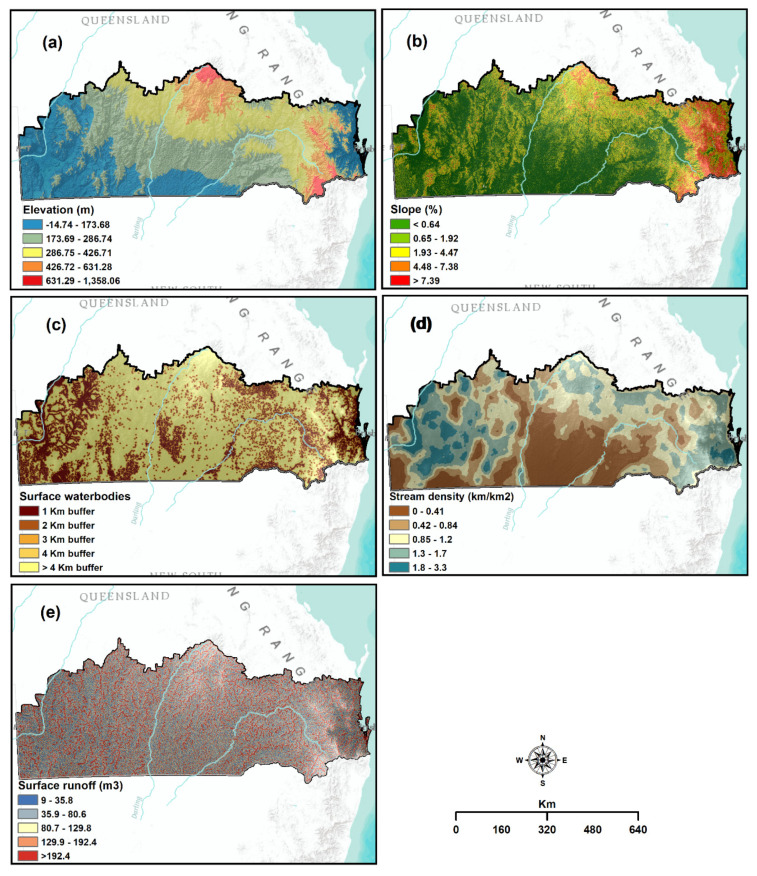
Thematic layers used for hydrological drought vulnerability: (**a**) elevation; (**b**) slope; (**c**) surface water bodies; (**d**) stream density; and (**e**) surface runoff.

The elevation and slope of an area could play an important role in hydrological drought vulnerability [30]. The vulnerability to drought is higher for regions that have a high elevation and steep slope, whereas lower vulnerability has prevailed in regions with low elevation and gentle slope [31,56]. A digital elevation model (DEM) was also used to produce an elevation spatial layer (Figure 4a). The DEM was collected from Queensland Spatial Catalogue–QSpatial at 90 m spatial resolution. The slope spatial layer was created using the data acquired from the Terrestrial Ecosystem Research Network (TERN) (Figure 4b).

Surface water bodies and stream density are also important factors in hydrological drought vulnerability assessment [15]. Regions close to water sources are less vulnerable to drought that are distant from water sources [3]. Similarly, areas with high stream density are assumed less vulnerable to drought due to their greater water contact than other areas [25,30]. The data of surface water bodies, including natural and man-made structures, were collected from the QLUMP for 2016, and the spatial analyst tool and Euclidean distance were used to create distance buffers from the surface water bodies to develop the spatial layer (Figure 4c). Contrarily, streamline data from Geoscience Australia were used to generate stream density spatial layer at 90 m spatial resolution (Figure 4d). To create this layer, the stream density was calculated by employing the line density tool of ArcGIS software.

Surface runoff is one of the important variables for hydrological drought vulnerability assessment [58]. In this study, we estimated the surface runoff using geospatial data such as digital elevation model (DEM), Land use, soil, and precipitation data following the methods explained in Pal and Samanta [59] and Vojtek and Vojteková [60]. Primarily, weighted curve number was calculated for each sub basin using Equation (1):

(1) $$Weighted\;sub\;basin\;CN=\frac{CN_{1}\times A_{1}+CN_{2}\times A_{2}\ldots +CN_{n}\times A_{n}}{A_{1}+A_{2}+\ldots +A_{n}}$$

where, $CN_{1}$, $CN_{2}\;$… $CN_{n}$ are the curve numbers for different land uses and treatment, and hydrologic soil groups present in the sub-basin of the total river basin. $A_{1}$, $A_{2},\;\ldots \;A_{n}$ are its respective sub-basin areas.

The weighted *CN* for the whole basin is calculated by using the Equation (2):

(2) $$Weighted\;CN=\frac{CN\;of\;sub-watershed\times it\;area}{Total\;area\;of\;watershed}$$

Subsequently, potential retention capacity *P* was calculated following Equation (3):

(3) $$P=25.4\;\left(\frac{1000}{CN}-10\right)$$

The runoff depth was calculated using Equation (4):

(4) $$H_{0}=\frac{{\left(H_{p}-0.2P\right)}^{2}}{\left(H_{p}+0.8P\right)}$$

In addition to the potential retention of catchment (*P*), the formula also contains the value of average daily rainfall with N-year return period ($H_{p}$).

The next step was to create a raster of contributing areas ($S_{p}$) in the catchment where each cell is inserted the number of connected cells in the direction of flow above this cell and their size is then calculated. Equation (5) was used for the calculation of contributing areas ($S_{p}$) where the cell size has a value of 90 m^2^:

(5) $$S_{p}=\mathrm{accumulation}\;\mathrm{raster}\;\times \mathrm{cell}\;\mathrm{size}/1,000,000$$

Finally, the surface runoff was calculated based on Equation (6):

(6) $$O_{p}=H_{0}\times S_{p}\times 1000$$

#### 3.2.3. Agricultural Drought Vulnerability Mapping Criteria

Agricultural drought occurs when water is insufficient for crop growth and production [15,30]. Land use/land cover (LULC), plant available water capacity (PAWC), soil depth and percentage of soil moisture were selected as relevant criteria for mapping agricultural drought vulnerability.

**Figure 5:**
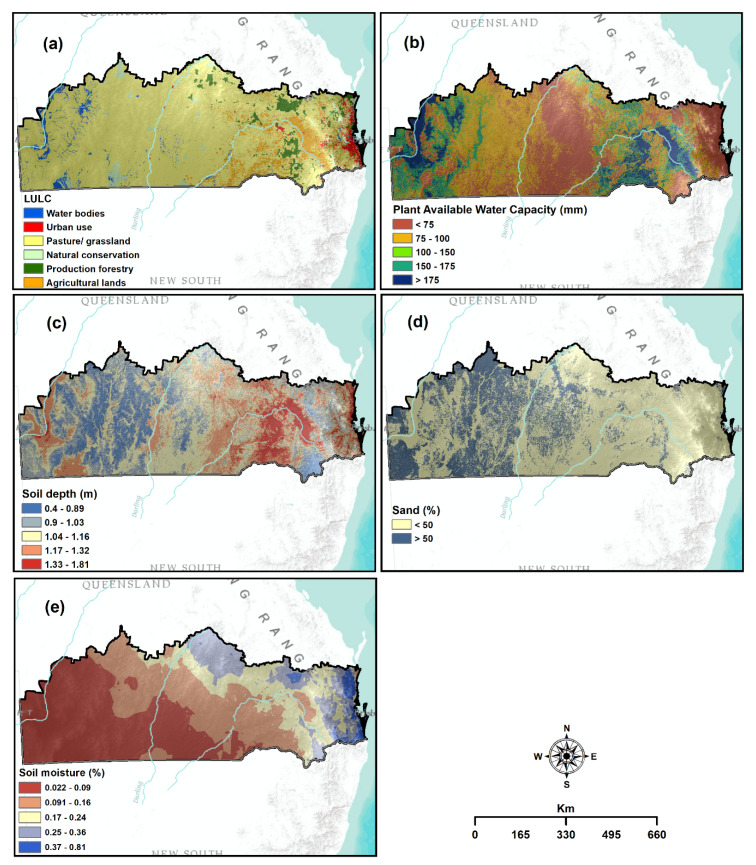
Thematic layers used for agricultural drought vulnerability: (**a**) LULC; (**b**) PAWC; (**c**) soil depth; (**d**) sand percentage; and (**e**) soil moisture.

The LULC is an important criterion for assessing agricultural drought vulnerability because it serves as a driving force behind water demand [61]. In this study, the 72 Sentinel imagery (10 m spatial resolution) was used to prepare the LULC spatial layer (Figure 5a). The Sen-2 Cor toolbox of SNAP software was used to perform the entire pre-processing task of sentinel images. The hybrid classification approach, a combination of un-supervised and supervised (maximum likelihood algorithm) classification techniques, was followed to classify eight different LULC classes: production forestry, water bodies, urban use, pasture/grassland, natural conservation and agricultural land [20]. Accuracy assessment of classified images was performed using Kappa accuracy assessment technique [62]. High-resolution Google Earth images were used to generate validation samples that correspond to the same period as these Sentinel images. In total, 1500 random samples with 200 samples for each LULC category were collected using stratified random sampling. The overall accuracy and Kappa coefficients of the produced LULC map were found 89% and 87%, respectively.

PAWC extremely influences agricultural drought vulnerability. PAWC indicates the difference between water content within field capacity and permanent wilting point [20]. When the level of PAWC decreases, drought vulnerability increases [63]. Thus, a PAWC spatial layer utilising the data acquired from the Australian NAMS was produced in this study (Figure 5b).

Soil depth and soil texture (sand) are important factors for agricultural drought vulnerability assessment [64]. The thickness of the soil materials is attributed to soil depth that influences plant growth, providing water and nutrients [25]. Areas that have higher soil depth are less vulnerable to drought than those with shallow soil depth because of their higher capacity to preserve and provide moisture to plants for growing [20]. Soil depth spatial layer was created using data from TERN (Figure 5c). Similarly, soil texture controls the water holding capacity in the soil. Therefore, proneness to drought is higher for areas characterised by high percentages of sand [30]. Therefore, sandy soil data from TERN for 2014 was used to generate a sand percentage spatial layer (Figure 5d). The detailed processing of these data was explained by Dayal, et al. [20].

Soil moisture is another vital criterion influencing agricultural drought vulnerability [64]. The area with low soil moisture content is highly vulnerable to agricultural drought than those with high soil moisture content [65]. Hence, a soil moisture spatial layer was prepared by applying soil moisture data at 90 m spatial resolution from 2014–2018. The data were obtained in NetCDF format from the Bureau of Meteorology, Australia, and then converted into a raster format. Finally, a single spatial layer averaging all year values was created (Figure 5e).

### 3.3. Standardisation Criteria Layers and Alternative Ranking

Vulnerability ratings were assigned for all alternatives of spatial criteria layers following a numerical ranking scheme. The numerical ranking scheme used values from 1 to 5, where 1 represents extremely low vulnerability, and 5 has extremely high vulnerability. The value of rank for each alternative was assigned to the contribution of alternatives to drought vulnerability (Table 2). The corresponding alternative ranking values were standardised in a uniform range from 0–1 using Equation (7). The standardisation was required to integrate all criteria alternatives uniformly and facilitate the application of the spatial multi-criteria decision-making approach:

(7) $$p=\frac{x\;-\;\min}{\max\;-\;\min}$$

where, p specifies the standardised value; min and max refer to the minimum and maximum values of each dataset, respectively; x indicates the cell value. 

### 3.4. Weighting the Criteria Using FAHP

Multi-criteria weighting technique FAHP was used in this study given that FAHP is a proven technique to manage uncertainties that arise by giving the preferences in the multi-criteria weighting decision-making process [66,67]. Various FAHP approaches have been applied in several studies [38,39,68]. Moreover, an integrated FAHP approach was used this study, which was developed by Chang [68]. A triangular fuzzy number (TFN) was also used with this approach to simplify the pair-wise comparison process. The five-step FAHP method [68], which was followed here for weighting the multi-criteria, is described below: 

*Step 1*: the criteria linked to the drought vulnerability assessments were chosen.

*Step 2*: pairwise comparison matrices were prepared following the opinions of six experts. All of the experts had considerable experience in drought research, factors liable for droughts and their influence on the study site. They were selected from academic, government, and non-government research institutions. The experts provided their opinions considering the relative importance of chosen criteria. A geometric mean method was followed to incorporate the expert’s opinions as follows:

(8) R=(a,b,c),K=1,2,….,K(R:triangular fuzzy member and K: no. of DMs

where a=(a1×a2×…×ak)1k, b=(b1×b2×…×bk)1k, c=(c1×c2×…×ck)1k**.**

*Step 3*: pairwise comparison matrixes were aggregated, and the judgments were synthesised to develop a set of overall priorities for the hierarchy.

*Step 4*: the consistency of experts’ opinions in pairwise metrices was justified by calculating the consistency ratio (CR). The rating is considered true if the consistency ratio is equal to or less than 0.1. To calculate the CR, was adopted:

CR= Consistency Index/Random Index,

where, the random index (RI) was calculated on the basis of the following the matrix order (*n*) developed by Saaty [69]. Moreover, consistency index (CI) was measured by the following equation:

$$\mathrm{CI}=(\lambda_{max}-n)/\left(n-1\right),$$

where, λ*_max_* and *n* present the largest eigenvalue and order of a matrix, respectively [70]. To maintain the consistency in the pairwise comparisons, the CR value should be lower than 0.10 [71]. The calculated criteria and drought category weights from the developed matrices and CR values are presented in Table 3.

*Step 5*: Pairwise matrix criteria weights were transformed into linguistic variables following Table A1 in Appendix A. The priority weights were determined using Chang [69]’s method (Table 3).

### 3.5. Overall Vulnerability Assessment

Adopting the weighted overlay approach, the drought vulnerability index was prepared for each drought type, incorporating their related criteria weights. The overall drought vulnerability index combining all categories of drought indices was then developed by reapplying the weighted overlay approach using their respective weights. The index values of each drought type and overall drought were then standardised using Equation (1) to convert into a uniform range from 0–1. Afterwards, the standardised indices values were classified into five categories (i.e., normal, mild, moderate, severe and extreme vulnerability) to create maps of meteorological, hydrological, agricultural and overall drought vulnerability. The natural breaks classification technique was applied to classify these maps because using this technique is consistent and efficient in presenting the spatial pattern of drought in the study site [50].

### 3.6. Validating Drought Vulnerability Maps

To validate the result of the produced three drought vulnerability maps, the ROC and AUC approach was adopted. This is a classical way to evaluate the susceptibility and vulnerability model accuracy. This approach is the most suitable technique to evaluate the effectiveness of deterministic and probabilistic justification [72]. Therefore, the ROC-AUC technique was used to verify the produced three drought vulnerability maps following Equation (5), where, *TP* is true positive, *TN* is true negative, *P* is positive and *N* is negative:

(9) $$\frac{\sum TP+\sum TN}{P+N}$$

In the present study, the prediction rate curve was generated using soil moisture data, rainfall data and surface water bodies data for agriculture, meteorological and hydrological drought, respectively. The relative departure of rainfall (RDR) is a common hazard index used for identifying meteorological drought [2]. Similarly, validation of agricultural drought vulnerability map applying soil moisture data is appropriate through the relative departure of soil moisture (RDMS) given that the moisture content works as a crucial indicator of agricultural droughts [15]. However, no appropriate method to validate hydrological drought was found; therefore, the relative departure of surface water bodies (RDSW)was adopted. In the RDSW, the water bodies during the drought period and non-drought period were calculated.

A few steps were followed to perform the procedure. At first, the soil moisture dataset (2014–2018) from the Australian Government, Bureau of Meteorology, the precipitation data (1970–2018) and the surface water data (2013 and 2018) were acquired. Afterwards, following the methods by Rahmati, et al. [2], the *RDR*, *RDSM* and *RDSW* were calculated and then an integrated drought inventory map was generated employing Equations (6)–(8):

(10) $$RDR=\frac{x_{i}-{\;\bar{x}}_{i}}{{\bar{x}}_{i}}\times 100\;$$

(11) $$RDSM=\frac{S_{i}-{\bar{S}}_{i}}{{\bar{S}}_{i}}\times 100\;$$

(12) $$RDSW=\frac{z_{i}-{\bar{z}}_{i}}{{\bar{z}}_{i}}\times 100\;$$

where, $x_{i}$ indicates rainfall for the given year (2013) and ${\bar{x}}_{i}$ refers mean annual rainfall over the base period (1970–2018), $S_{i}$ specifies annual soil moisture for 2013 (Queensland had the driest year in the history) and ${\bar{S}}_{i}$ indicates mean annual soil moisture between 2005 and 2019, $z_{i}$ represents the surface water bodies for 2013 and ${\bar{z}}_{i}$ denotes surface water bodies for 2018. In the next step, the values of RDR, RDSM and RDSW were standardised into a 0–1 scale employing a fuzzy logic operation process. Then, 0.5 threshold was used for the RDR (i.e., *RDR* > 0.5), RDSM (i.e., *RDSM* > 0.5) and RDSW (i.e., *RDSW* > 0.5) to determine drought-affected locations for meteorological, hydrological and agricultural droughts in the study sites, respectively. The whole validation procedure was adopted from Rahmati, et al. [2] and Rahmati, et al. [73]. The authors employed a threshold of 0.5 for a similar environment (southern Queensland). Following Rahmati, et al. [2] and Rahmati, et al. [73], we employed >0.5 as a standard threshold value. Next, 984 drought locations (317 for meteorological, 347 for agricultural and 320 for hydrological) were randomly chosen to validate the prepared drought vulnerability maps where the validation data represent 100% of the drought location points.

## 4. Results and Discussion

### 4.1. Meteorological Drought Vulnerability Mapping

**Figure 6:**
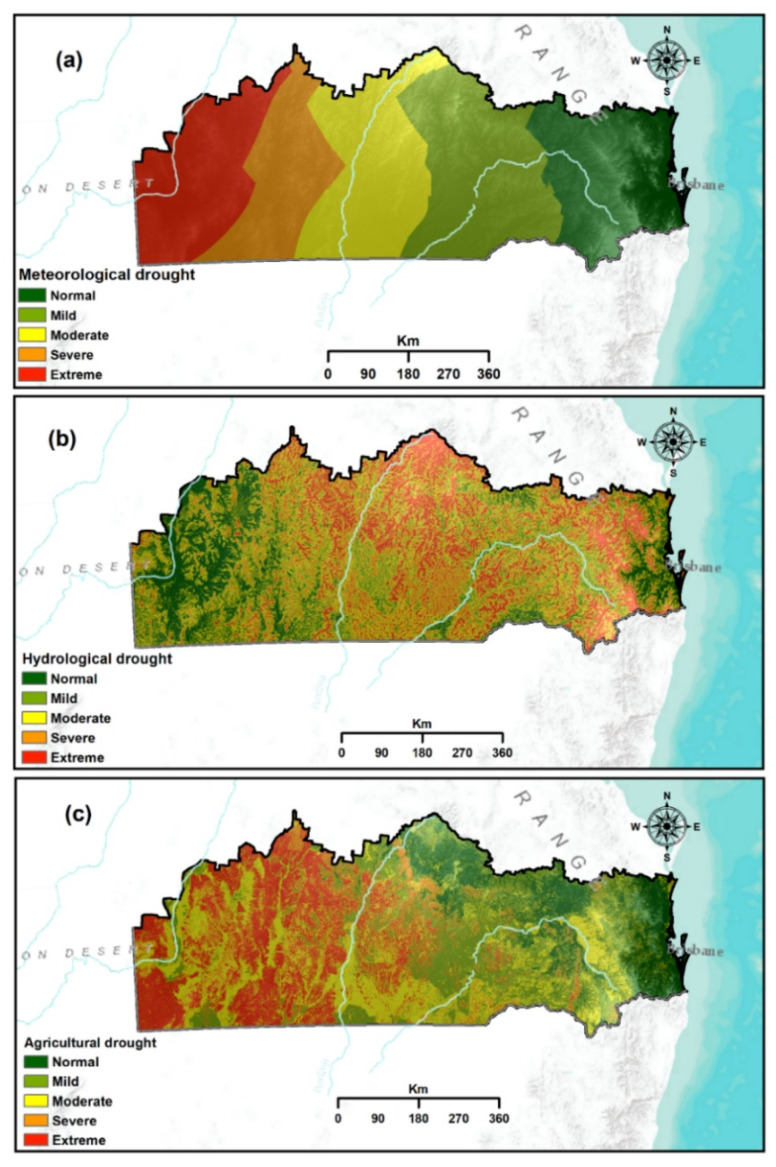
Overall vulnerability map indicating the spatial footprint and vulnerability to drought: (**a**) meteorological drought; (**b**) hydrological drought; and (**c**) agricultural drought.

To validate the result of the produced three drought vulnerability maps, the ROC and AUC approach are presented in Figure 6a reveal that the severe and extreme drought vulnerability area covers roughly 14.98% (65,076.8 km^2^) and 18.88% (82,009.8 km^2^) of the study area, accordingly. 

Around 21.38% (92,875.5 km^2^) of the study region was classified as a moderately vulnerable zone. These moderate to extreme drought vulnerable zones are found mostly in the northern, northwestern and southwestern parts of the study region, particularly Bulloo, Quielpie, Murweh and Parro. Less rainfall, high temperatures and high evaporation prevail in these regions. Meanwhile, around 44.77% (194,479 km^2^) of the study site are classified as normal to mild drought vulnerability zones. Most of these areas are located in the eastern, northeastern and southeastern portions of the studied region, which have low rainfall, low temperature and low evaporation.

### 4.2. Hydrological Drought Vulnerability Mapping

Figure 6b demonstrates the spatial distribution and pattern of hydrological drought vulnerability of the study region. The extreme drought vulnerability category accounts for approximately 15.4% (63,167.04 km^2^) of the study region, which is lower than other drought vulnerability categories. The area labelled as moderate to extreme drought vulnerable region is 68.94% (289,574.51 km^2^). These highly vulnerable regions are found in the central, northeastern and southeastern portions of the study site, particularly Murweh, Maranoa, Balonee, Western Downs and Goodniwindi. The high elevation, steep slope and less surface water bodies of these regions are considered factors that cause high vulnerability to hydrological drought. Conversely, places that have normal to mild drought vulnerability cover 9.47% (39,774.13 km^2^) and 21.57% (90,628.34 km^2^), respectively, located mostly in western, northwestern, southwestern and eastern portions of the study region.

### 4.3. Agricultural Drought Vulnerability Mapping

Figure 6c exhibits the spatial distribution and pattern of agricultural drought vulnerability of the study area. The extreme and severe agricultural drought vulnerable categories constitute 91,674.87 and 44,122.4 km^2^ of the study area, accordingly and cover 31.26% of the study site (Table 4). These highly vulnerable areas are found mostly in the western, northwestern and southwestern portions of the study region covering Bulloo, Quilpie and Paroo. The areas classified as moderately vulnerable to agricultural drought represent 28.01% (121,665.1 km^2^) of the total study site. These classified areas are scattered mostly in the central and south-eastern portions of the study site, particularly Balonne, Maranoa, Goondiwindi, Western Downs and Lockyer valley. All these moderate to extremely vulnerable regions are characterised by expansive agricultural lands, shallow soil depth, sandy and less PAWC. Areas classified as mild to normal vulnerability to agricultural drought comprise 26.22% (1,139,906.9 km^2^) and 13.83% (60,076.74 km^2^) of the study area, accordingly. Land use patterns, high soil depth and high PAWC are observed in areas with low agricultural drought vulnerability.

### 4.4. Overall Drought Vulnerability Mapping

**Figure 7:**
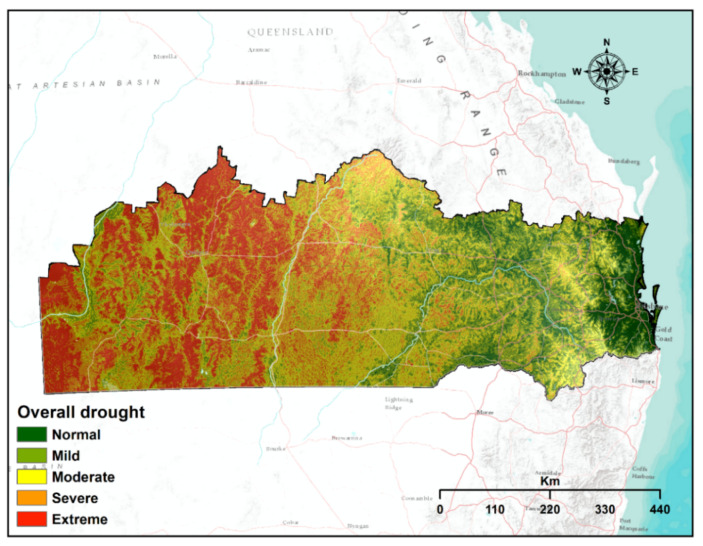
Overall vulnerability map indicating the spatial footprint and vulnerability to overall drought.

Figure 7 presents the overall vulnerability to drought in the study area. The extreme and severe overall drought vulnerable categories in the study regions cover 16.86% (70,824.2 km^2^) and 100,997.17% (24.4%), respectively, representing approximately 40.6% of the total study regions (Table 4). These regions are observed mostly in the western, northwestern, southwestern and some middle portions, specifically, Bulloo, Quilpie, Parro, Murweh, Balonne and Maranoa. Areas classified as moderately vulnerable to overall drought comprise 21.49% (93,357.94 km^2^) of the total study regions. These regions are concentrated mostly in the eastern, northeastern and southeastern portions of the study sites, covering Goodniwindi, Western Downs, Lockyer valley, Western Downs and Toowoomba. Meanwhile, normal and mild drought areas encompass approximately 7.83% (32,898.09 km^2^) and 20.81% (87,403.22 km^2^) of the study regions, accordingly. These regions are scattered in portions of the eastern and northern parts, especially areas towards the coastline. Overall, nearly the total area, except for several areas of the eastern and northeastern regions, can be labelled as drought prone.

### 4.5. Validation of Drought Vulnerability Maps

**Figure 8:**
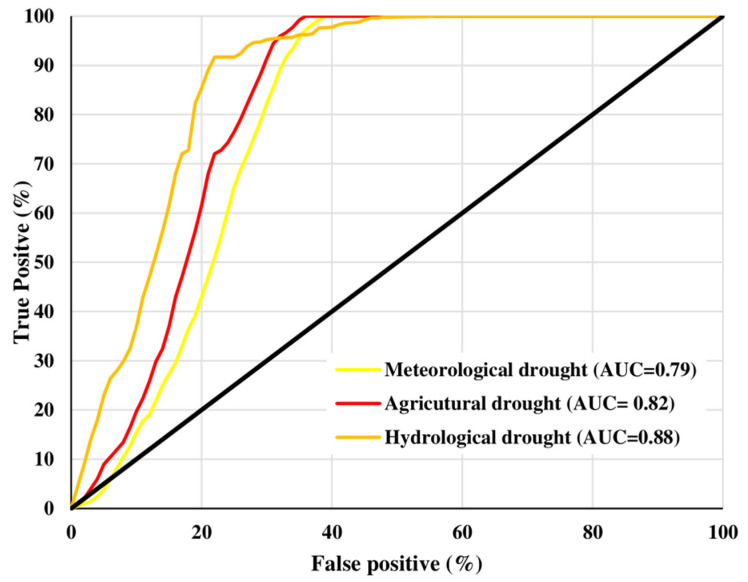
Area under curve (AUC) for different types of drought maps.

Figure 8 demonstrates the prediction rate curves, providing model efficiency applied in the current study. The AUC of the prediction rate for the meteorological, agricultural and hydrological vulnerability maps were 0.79, 0.82 and 0.88, respectively, explaining 79.3%, 82.7% and 86.8% AUC prediction accuracy of the tested model. The values of AUC vary between 0.5 and 0.1, and value close to 1 shows higher accuracy [72]. Consequently, AUC values of the prediction rate (79.3%, 82.7% and 88.8%) of this study present a convincing outcome of our prepared and tested drought vulnerability mapping approach. Moreover, our findings corroborated with the existing studies applied in a similar environment [2,21,67,74]. For instance, Rahmati, et al. [73] and Rahmati, et al. [2] validated their models for agricultural and meteorological droughts. A similar method was also applied by Dayal, et al. [20] for validating drought risk. Later Rahmati, et al. [73] and Rahmati, et al. [2] modified the method to apply it only for specific drought hazards, such as meteorological or hydrological. Rahmati, et al. [73], Rahmati, et al. [2] and Dayal, et al. [20] tested their models in south-east Queensland, Australia whilst we targeted the entire southern Queensland.

## 5. Discussion

In the recent past, drought has been one of the major concerns for Australia. Due to recurrent drought incidents, crop production, livestock farming, the river flows, water-dependent ecosystems, rural and urban communities of Australia have been affected severely [17,74]. Several studies anticipated further increase in trend of drought for Australia in more severe scale [75,76,77,78]. Therefore, this study designed to develop a comprehensive drought vulnerability map that combines three common drought components by applying geospatial techniques and to assess the spatial extent of drought vulnerability for southern Queensland. We selected the relevant variables to assess the particular drought type vulnerability which are influencing the relevant drought category vulnerability following the methodology of several published articles in renowned journals [25,26,34,35,55]. We believe that chosen variables had direct impacts on the particular drought hazard and were effective in the assessment of drought vulnerability. Moreover, we mostly focused on physical aspects of drought vulnerability in this study.

The findings demonstrate that extreme and severe overall drought-vulnerable categories in the study regions cover 20.1% and 20.5%, respectively, representing approximately 40.6% of the total study regions. These regions are observed mostly in the western, northwestern, southwestern and some middle portions, specifically, Bulloo, Quilpie, Parro, Murweh, Balonne and Maranoa. Our findings of meteorological, agricultural, and overall drought corroborated with existing relevant studies of Rahmati, et al. [2], Dayal, et al. [20], Rahmati, et al. [73] and Kiem, et al. [79]. These studies focused on the southeastern part of Queensland. Similarities were observed between vulnerability index by Dayal, et al. [20] and the findings of the current overall drought vulnerability. Such resemblance is also found between Rahmati, et al. [2] and Rahmati, et al. [73] with the current findings from agricultural vulnerability index. The overall drought vulnerability is the final outcome of individual drought indices such as meteorological, agricultural, and hydrological drought. These individual indices were validated through ROC technique and all the indices have an accuracy close to 80%. With an acceptable level of accuracy, all the drought indices reveal appropriateness of applied models first applied by Hoque, et al. [53].

This study also has several drawbacks. For example, only meteorological, hydrological and agricultural categories were integrated into this study as categories of drought for vulnerability assessment. We were unable to include socio-economic drought effects. Although we integrate three types of drought, the time scales were not same which can deliver potential error. Managing datasets at similar time scales was challenging. Similarly, application of the annual time step index was underestimated. Moreover, the study benefitted from high-resolution datasets for preparing some criteria layers (e.g., elevation, LULC, and slope). Most data were used in this study were at low spatial resolution (90 m). As a result, our final outputs were mapped at only 90 m spatial resolution. High spatial resolution maps can improve the accuracy and visualisation of the findings. Historical drought records were used to validate our final outputs because we could not collect field data due to time constraint and funding issues. In terms of meteorological drought, we rely on the meteorological stations that are capable of providing data without year gaps, thus the meteorological stations do not cover the entire study area. However, meteorological stations that cover the whole study area could provide a better outcome. Further, the weights determined by FAHP technique is basically based on the experience of the experts. Due to time frame and funding, we are unable to consider other methods for this study. The application of robust machine learning method is planned in our future work. Further, it would be better to include the drought impact sectors in the drought vulnerability assessment. However, due to time frame and funding, we are unable to consider the drought impact sectors at this moment. It will be our future effort. We attempted to validate the final vulnerability map using NDWI. However, our validation method requires datasets for 2013 which is one of the driest years in history. We already used Sentinel-2 for generating LULC and to maintain consistency Sentinel-2 is also required for NDWI. Unfortunately, Sentinel-2 datasets are not freely available for 2013. Further, we also require time series datasets for computing mean NDWI between 2013 and 2018 as we did for other drought validation. Due to the limited timeframe, it is not possible for us to deal with 60+ satellite images for each year. It will be our future effort. Along with the above drawbacks, future studies can also consider relevant climate change factors that influence each type of drought. Despite these limitations, the findings of this study are still useful in the development of drought mitigation measures to minimise drought losses and its effects on agricultural, socio-economic activities and environments.

## 6. Conclusions

The current study aimed to develop a comprehensive drought vulnerability map and assess the spatial extent of the overall vulnerability of droughts for the southern Queensland regions of Australia. For the first time, the three categories of drought with their relevant criteria are combined to produce reliable outputs in the study region. To this end, the preparation, processing and integration of criteria and drought types were performed using geospatial techniques and FAHP. The overall drought vulnerability map was verified successfully by using the ROC and AUC approach. The overall drought vulnerability map demonstrates severe to extreme drought vulnerability for Bulloo, Quilpie, Paroo, Murweh, Balonne and Maranoa areas of the study site. Except for several areas in the eastern (i.e., Gympie, Noosa, Somerset, Ipswich, Logan and Scenic Rim) and northeastern regions (i.e., South Burnett and Southern Downs), all areas of the study site are under mild-to-moderate drought vulnerable areas. The results can assist concerned authorities and policymakers in visualising drought vulnerability and its level of intensity and developing proactive drought mitigation strategies. 

## Figures and Tables

**Table 1:** The used datasets for drought vulnerability assessment.

Criteria	Types	Source	Period
Land use and land cover (LULC)	Sentinel-2 (10 m resolution)	USGS Earth Explorer (https://earthexplorer.usgs.gov/), accessed on 4 April 2018	2018 (January–February)
Elevation	3-s DEM data (90 m resolution)	Queensland Spatial Catalogue–QSpatial	2000
Slope	In percentages (90 m resolution)	Terrestrial Ecosystem Research Network (TERN)	2000
Surface water bodies	QSpatial data (90 m resolution)	Queensland Land Use Mapping Program (QLUMP)	2016
Plant available water capacity (PAWC)	90 m resolution	National Agricultural Monitoring system (NAMS; http://www.nams.gov.au), accessed on 3 February 2018	2014
Soil depth, Sand percentage	90 m resolution	TERN	2014
Soil Moisture	90 m resolution (NetCDF format)	Australian Government, Bureau of Meteorology (http://www.bom.gov.au), accessed on 1 March 2018	2014–2018
Stream density	Polyline	Geoscience Australia (https://www.ga.gov.au/), accessed on 7 May 2018	2016
Mean annual rainfall, mean annual maximum temperature, mean annual humidity	90 m resolution	Australian Government, Bureau of Meteorology (http://www.bom.gov.au), accessed on 10 March 2018	1970–2018
Mean annual evaporation	90 m resolution	Australian Government, Bureau of Meteorology (http://www.bom.gov.au), accessed on 9 March 2018	1975–2005

**Table 2:** Alternative ranking of each criterion following the influence to drought vulnerability.

Component	Criteria	Classes	Rank	Vulnerability
Meteorological drought	Mean annual maximum temperature (°C)	22.38–26.98	1	Very low
		26.99–27.86	2	Low
		27.87–28.32	3	Moderate
		28.33–29.04	4	High
		29.05–30.1	5	Very high
	Mean annual rainfall (mm)	639.38–1607.82	1	Very low
		535.81–639.37	2	Low
		411.51–535.8	3	Moderate
		307.94–411.5	4	High
		287.21–307.33	5	Very high
	Mean annual humidity (%)	63.38–75.99	1	Very low
		56.82–63.37	2	Low
		49.99–56.81	3	Moderate
		46.52–49.98	4	High
		43.16–46.51	5	Very high
	Mean annual evaporation (mm)	1600–2000	1	Very low
		2000–2400	2	Low
		2400–2800	3	Moderate
		2800–3200	4	High
		>3200	5	Very high
Hydrological drought	Elevation (m)	<173.68	1	Very low
		173.69–286.74	2	Low
		286.75–426.71	3	Moderate
		426.72–631.28	4	High
		>631.28	5	Very high
	Slope (%)	0–0.64	1	Very low
		0.65–1.32	2	Low
		1.33–4.47	3	Moderate
		4.48–7.38	4	High
		>7.38	5	Very high
	Surface water bodies (km)	1	1	Very low
		2	2	Low
		3	3	Moderate
		4	4	High
		5	5	Very high
	Stream density (km/km2)	1.8–3.3	1	Very low
		1.3–1.7	2	Low
		0.85–1.2	3	Moderate
		0.42–0.84	4	High
		0–0.41	5	Very high
	Surface runoff (m^3^)	9–35.8	1	Very low
		35.9–80.6	2	Low
		80.7–129.8	3	Moderate
		129.9–192.4	4	High
		>192.4	5	Very high
Agricultural drought	LULC	Water bodies, natural conservation	1	Very low
		Production forestry	2	Low
		Pasture/grassland	3	Moderate
		Urban use	4	High
		Agricultural lands	5	Very high
	PAWC (mm)	>175	1	Very low
		150–175	2	Low
		100–150	3	Moderate
		75–100	4	High
		<75	5	Very high
	Soil depth (m)	1.33–1.81	1	Very low
		1.17–1.32	2	Low
		1.04–1.16	3	Moderate
		0.9–1.03	4	High
		0.4–0.89	5	Very high
	Soil moisture (%)	0.37–0.81	1	Very low
		0.25–0.36	2	Low
		0.17–0.24	3	Moderate
		0.091–0.16	4	High
		0.022–0.09	5	Very high
	Sand (%)	<50	2	Low
		>50	4	High

**Table 3:** Criteria weights and CR values.

Component	Criteria	Weight
Meteorological drought	Mean annual maximum temperature	0.249
	Mean annual rainfall	0.305
	Mean annual humidity	0.209
	Mean annual evaporation	0.238
CR: 0.06		
Hydrological drought	Elevation	0.208
	Slope	0.140
	Surface water bodies	0.22
	Stream density	0.0.217
	Surface runoff	0.215
CR: 0.03		
Agricultural drought	LULC	0.140
	PAWC	0.220
	Soil depth	0.215
	Soil moisture	0.217
	Sand percentage	0.208
CR: 0.03		
Overall drought vulnerability	Meteorological drought	0.267
	Hydrological drought	0.390
	Agricultural drought	0.343
CR: 0.06		

**Table 4:** Area coverage of meteorological, hydrological, agricultural and overall drought vulnerability classes and share of drought events according to the defined drought vulnerability classes.

Vulnerability Class	Meteorological Drought		Hydrological Drought		Agricultural Drought		Overall Drought	
	Area (sq. km)	Area (%)	Area (sq. km)	Area (%)	Area (sq. km)	Area (%)	Area (sq. km)	Area (%)
Normal	87,926.82	20.24	39,774.13	9.47	60,076.74	13.83	32,898.09	7.83
Mild	106,551.73	24.53	90,628.34	21.57	113,906.86	26.22	87,403.22	20.81
Moderate	92,875.52	21.38	116,645.94	27.77	121,665.09	28.01	125,223.64	29.81
Severe	65,076.79	14.98	109,761.53	26.13	44,122.40	10.16	100,997.17	24.04
Extreme	82,009.77	18.88	63,167.04	15.04	91,674.87	21.10	70,824.2	16.86

## Data Availability

Raw data were generated at University of Technology Sydney, Australia. Derived data supporting the findings of this study are available from the corresponding author [Biswajeet Pradhan] on request.

## Appendix A

**Table A1:** Membership function of linguistic scale.

Linguistic Variable	Crisp Number	Triangular Fuzzy Numbers	Reciprocal Triangular Fuzzy Numbers
Equally strong	1	(1, 1, 1)	(1, 1, 1)
Moderately strong	3	(2, 3, 4)	(1/4, 1/3, 1/2)
Strong	5	(4, 5, 6)	(1/6, 1/5, 1/4)
Very strong	7	(6, 7, 8)	(1/8, 1/7, 1/6)
Extremely strong	9	(9, 9, 9)	(1/9, 1/9, 1/9)
Intermediate	2	(1, 2, 3)	(1/3, 1/2, 1)
	4	(3, 4, 5)	(1/5, 1/4, 1/3)
	6	(5, 6, 7)	(1/7, 1/6, 1/5)
	8	(7, 8, 9)	(1/9, 1/8, 1/7)
